# Helminths in the hygiene hypothesis: sooner or later?

**DOI:** 10.1111/cei.12353

**Published:** 2014-06-09

**Authors:** R M Maizels, H J McSorley, D J Smyth

**Affiliations:** Institute for Immunology and Infection Research, Centre for Immunity, Infection and Evolution, University of EdinburghEdinburgh, UK

**Keywords:** allergy, autoimmunity, immunoregulation, infection, therapy

## Abstract

There is increasing recognition that exposures to infectious agents evoke fundamental effects on the development and behaviour of the immune system. Moreover, where infections (especially parasitic infections) have declined, immune responses appear to be increasingly prone to hyperactivity. For example, epidemiological studies of parasite-endemic areas indicate that prenatal or early-life experience of infections can imprint an individual's immunological reactivity. However, the ability of helminths to dampen pathology in established inflammatory diseases implies that they can have therapeutic effects even if the immune system has developed in a low-infection setting. With recent investigations of how parasites are able to modulate host immune pathology at the level of individual parasite molecules and host cell populations, we are now able to dissect the nature of the host–parasite interaction at both the initiation and recall phases of the immune response. Thus the question remains – is the influence of parasites on immunity one that acts primarily in early life, and at initiation of the immune response, or in adulthood and when recall responses occur? In short, parasite immunosuppression – sooner or later?

OTHER ARTICLES PUBLISHED IN THIS REVIEW SERIES

*Lessons from helminth infections: ES-62 highlights new interventional approaches in rheumatoid arthritis. Clinical and Experimental Immunology 2014, 177: 13–23*.

*Microbial ‘old friends’, immunoregulation and socioeconomic status. Clinical and Experimental Immunology 2014, 177: 1–12*.

*Intestinal microbiota and faecal transplantation as treatment modality for insulin resistance and type 2 diabetes mellitus. Clinical and Experimental Immunology 2014, 177: 24–9*.

*The intestinal microbiome in type 1 diabetes. Clinical and Experimental Immunology 2014, 177: 30–7*.

## Introduction

The ability of infectious agents to regulate the immune system of their host is an increasingly fascinating topic. In particular, the question is often raised as to whether the ‘diseases of modernity’ (such as allergies, autoimmunity and the metabolic syndrome) are a consequence of our altered, and diminished, exposure to infectious diseases 1–3. Following a wide range of studies in humans and model systems, good evidence has now emerged that both microbes and macroparasites can sufficiently distract or depress immune reactivity to alleviate allergic and autoimmune pathologies 4–7.

The ‘hygiene hypothesis’ takes a number of forms which are not exclusive, but have yet to be articulated as a unifying concept. An inverse relationship between parasite infection and immune disorders was first suggested by Greenwood, who noted the low incidence of rheumatoid arthritis in West Africa 8, and then showed that mice and rats infected with rodent malaria were protected from autoimmune disease 9,10. Subsequently, the hygiene hypothesis became linked explicity to the setting of more developed countries when Strachan postulated that early-life exposure to common childhood infections protected younger siblings in larger families from developing allergies such as hay fever 11,12. At that time, soon after the emergence of the paradigm of opposing T helper type 1 (Th1) and Th2 arms of the immune system 13, this finding was interpreted as Th1-promoting viral and bacterial infections ‘educating’ the young immune system away from excessive and allergy-promoting Th2 responses. The core concept of infections imprinting the developing immune system has become embedded in most versions of the hygiene hypothesis, but the mechanistic explanation of opposing Th1/Th2 lineages has, over time, proved untenable.

In contrast to the Th2-mediated allergies [mediated through immunoglobulin (Ig)E, mast cells and eosinophils], other modern maladies such as type I diabetes, multiple sclerosis and Crohn's disease are driven by Th1 responses, or by the more recently defined Th17 cells 14. Critically, these Th1/17 conditions have been increasing in prevalence in high-income countries as sharply as Th2-dependent allergies. For example, the incidence of type I diabetes is increasing year-on-year at the rate of 3·4% 15, in tandem with the rise in asthma 16. Tellingly, the age of diabetes onset has become significantly younger, indicating that this disease is gaining in force within the population. Similar patterns have been observed for multiple sclerosis and Crohn's disease, and it is difficult to reconcile the accentuation of these diseases with reduced exposure to Th1-stimulating microbes in early life.

## Globalization of the hygiene hypothesis

A new perspective on the original hygiene hypothesis was introduced by a study of children in Gabon. In this West African country there is a high prevalence of schistosomiasis, a helminth worm disease which drives a strong Th2 phenotype in the infected host 17. Remarkably, schoolchildren carrying this infection showed lower levels of allergic reactivity than their uninfected classmates, although both the parasite and the allergen evoke Th2 responsiveness 18. A key pointer from this study was that the infected children generated high levels of an immunosuppressive cytokine, interleukin (IL)-10, when their peripheral T cells were challenged with antigen from the *Schistosoma* parasite. Numerous other studies, such as those in Brazilian children infected with schistosomes 19 and Ecuadorians with soil-transmitted nematodes 20, support the conclusion that helminth infections in many – but not all 21,22 – settings are associated with suppression of allergic reactivity.

IL-10 is one of two central mediators, together with transforming growth factor (TGF)-β, which act to dampen and down-regulate the immune system 23,24. While each can be produced by a range of cell types, they are particularly associated with a T cell subset that was defined between 1995 and 2000, the regulatory T cell (T_reg_) 25,26. Significantly, T_regs_ and their products (including IL-10 and TGF-β) were found to control both Th2 allergies 27,28 and the Th1/17 suite of autoimmune and inflammatory pathologies 29. Hence, the hygiene hypothesis has been modified and updated to posit that infections, and infection experience, may set the balance between T_regs_, on one side, and active Th1, Th2 and Th17 populations on the other 1,6,30,31.

This emerging paradigm has been strengthened by a study of multiple sclerosis patients in Argentina, 12 of whom were found to have adventiously acquired asymptomatic gastrointestinal helminth infections. All 12 remained in remission for 5 years, while uninfected patients with similar disease severity at the outset of the study suffered multiple relapses 32. Infected patients showed strong IL-10 and TGF-β responses, unlike the relapsing uninfected individuals, but production of these cytokines declined in four patients subsequently given anthelminthic treatment who went on to develop exacerbation of disease 33. In a related example, an ulcerative colitis patient who deliberately self-infected with the human whipworm, *Trichuris trichiura*, reported significant alleviation of pathology 34. These reports have fuelled greater interest in the possibilities of therapy of inflammatory disorders with live helminths, or with products derived from these parasites, as discussed further below.

Other key studies have reported that anthelmintic treatment of children in parasite-endemic countries increases their allergic sensitivity, providing a causal link between helminth infection and protection of allergy 35. Although autoimmune disease has a relatively low incidence in these countries, measurements of anti-nuclear autoantibody in serum (a precursor but not a determinant of overt autoimmunity) showed reduced reactivity in schistosome-infected individuals and an increase following curative drug therapy 36.

Reports such as these have taken the hygiene hypothesis onto a global stage, and highlighted the contrast in immunological status and reactivity between the affluent developed world and the low-income countries in which helminth parasites are still highly prevalent. Furthermore, although more than 25% of the world's populations are infected with helminth parasites 37, it should be remembered that until the last century most humans would have frequently carried helminth parasites and that our immune system has intimately co-evolved with these organisms, a point we will return to subsequently.

## Helminths and regulation of the immune system

The finding that helminth infection is associated with reduced allergic reactivity chimed with a range of immunological studies on both mice and humans which demonstrated enhanced activity of T_regs_ in infected hosts. Humans carrying long-lived chronic infections with schistosomes or mosquito-borne filarial nematodes displayed a clear phenotype of immunological down-regulation, with high levels of IL-10 38 and stronger suppressive activity of T_regs_ 39. In particular, asymptomatic carriers who were effectively immunologically tolerant to the parasite presented a ‘modified’ Th2 in which IL-4 is produced but the pro-eosinophilic cytokine IL-5 is suppressed 40. However, in cases which do not tolerate helminths well, and which progress to various forms of pathology, T_regs_ are deficient and Th1/17 dominates 41.

The implication that the ability of helminths to suppress immunopathologies is mediated by T_regs_ has been validated in experimental animals. Mice infected with the intestinal nematode *Heligmosomoides polygyrus* show expanded T_reg_ numbers and function 42–45 and are resistant to allergic pathology in mouse models 46. Moreover, protection against allergy can be transferred by T_regs_ from an infected animal into uninfected but allergen-sensitized hosts 46,47. Just as humans appear to be protected from autoimmunity as well as allergy by some helminth infections, so too do mice show diminished levels of colitis 48,49 and type I diabetes 50–52 when carrying schistosome or intestinal nematode infestations.

Helminths are thought to promote T_regs_ to prolong their own survival in the host by defusing key elements of the immune system that would otherwise attack them 53. To test this supposition, strategies to experimentally deplete T_regs_ in helminth-infected mice have been studied, showing more rapid parasite killing following depletion 54,55. In addition, T_reg_ depletion can result in more severe pathology resulting directly from the presence of helminth worms in the intestinal tract 56,57. As well as T_regs_, however, helminths can drive regulatory B cell populations 58–60, natural killer T cells 61,62 and suppressive macrophage responses 63,64, each contributing to a profoundly down-modulated state.

## Early in life

The importance of early-life exposure in defining the set-point of immunological reactivity of the individual is now widely recognized 65. The window of sensitivity extends prenatally to the developing foetus, as the propensity of the newborn to develop allergic eczema is altered by maternal infection or exposure to probiotic bacteria. After parturition, exposure to microbial products from environmental organisms may also be sufficient to limit allergic reactivity 66.

Helminth parasites are certainly an important element of the environmental education imparted to the developing immune system. Offspring of mothers harbouring a filarial infection (*Wuchereria bancrofti*) during pregnancy were found in adulthood to be immunologically tolerant to the parasite 67. A broader effect, beyond antigen-specific tolerance, has now been reported in which helminth infection during pregnancy protects the newborn from allergic eczema in childhood 68,69. It will be fascinating to follow these cohorts of well-characterized children into adulthood, to evaluate more clearly the relative importance of preterm, infant and adult infectious exposure to the functioning of the immune system.

It is important to take note of the harmful, as well as beneficial, effects of helminth infection on the immune system in early life. In helminth-endemic areas of developing countries, vaccination is less effective than in the developed world, with the polio vaccine showing only 70–90% seroconversion rates in the former, compared to 97–100% in the latter 70. Furthermore, anthelmintic treatment of infected populations prior to vaccination leads to increased efficacy of the cholera and bacilli Calmette–Guérin (BCG) vaccines 71–73. To test if maternal imprinting was depressing early-life responsiveness, anthelmintic treatment of pregnant mothers was combined with subsequent testing of vaccination responses in their offspring. However, maternal treatment had no effect on vaccine efficacy in children 74, implying that infections of children themselves are most important for suppression of vaccine responses. In this same trial, however, levels of childhood eczema were again shown to increase in the children of anthelmintic-treated mothers, implying a maternal imprinting role on the development of allergic but not vaccine responses 75.

## Later in life – helminth therapy

A distinct strand of the hygiene hypothesis has developed which postulates that responses of the mature immune system can also be modulated significantly by infectious organisms, sufficiently so for particular commensal microbes or organisms of limited pathogenicity to be considered as potential therapies or prophylactics. Most of the experimental evidence for the hygiene hypothesis is also derived from infections of adult animals prior to or during the induction of an allergic or autoimmune inflammatory disease.

The principle that infectious agents can dampen inflammation in the adult immune system is currently being put to the test with a number of clinical trials utilizing live *Trichuris suis* or *Necator americanus* parasites for various indications, including Crohn's disease 76, coeliac disease 77 and multiple sclerosis 78, among others. Not all trials to date have proved successful, however, with treatment of allergic rhinitis found to be not beneficial 79,80, and most recently with Crohn's disease reporting benefits for some patients but failing to achieve statistical significance 81. Similarly, while the human hookworm *N. americanus* was found to dampen responses in coeliac disease patients, it did not achieve a level that significantly alleviated symptoms 77,82. While discouraging, these trials have been carried out on unstratified patient groups and there may well be subsets within each disease who are most likely to show improvement during infection. In the longer term, it may also prove desirable to identify immunosuppressive molecules from these parasites that can serve as future drug leads, thereby dissociating any beneficial properties of helminths from the need to impose active infection on patients.

An interesting parallel exists between the effect of helminth infection and specific immunotherapy (SIT) in which the patient is desensitized against particular allergens 83. In both, the immune response switches to the IgG4 isotype 84,85. Moreover, anthelmintic clearance of parasites results in rapid loss of IgG4 (and in other settings, exacerbation of allergic and autoimmune reactivity). These instances argue that the mode and degree of immune responsiveness remains relatively plastic in adult life, and that contemporary interactions with extant infection is as much or more influential on disease outcome than historical experience.

## Early and late in the immune response

Immunopathologies require both initiation and sustenance of the immune response against target allergens, autoantigens or bystander antigens (such as those expressed by harmless commensals). Hence, interventions may be either or both prophylactic or therapeutic, and the appropriate choice depends on correctly identifying the initiating factors (for example, antigen uptake) and those which maintain responsiveness and are responsible for progressive tissue damage (such as inflammatory cytokines).

**Fig. 1:**
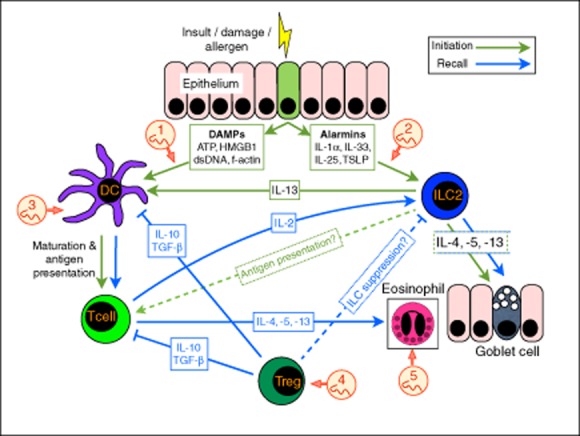
Sooner and later. The immediate response to immunological insult (in the form of injury, allergens or other toxic products) that causes epithelial cell stress and death, is the release of damage-associated molecular patterns (DAMPs), such as adenosine triphosphate (ATP), dsDNA, high-mobility group box 1 (HMGB1) and f-actin. Concurrently with, or consequent to DAMP release, the alarmin cytokines interleukin (IL)-1α, IL-33, IL-25 and thymic stromal lymphopoietin (TSLP) are induced. Both DAMPs and alarmins result in activation of dendritic cells (DCs) and group 2 innate lymphoid cells (ILC2). The responses occurring soon after immune stimulus are coloured green. ILC2 produce the type 2 cytokines interleukin (IL)-4, IL-5 and IL-13, resulting in subsequent expansion of effector cell populations such as eosinophils and goblet cells which can occur at both early (green arrows) and late (blue arrows) phases. ILC2-derived IL-13 also induces maturation and migration of DCs to the draining lymph node, where DCs present antigen to naive T cells. Although ILC2 express major histocompatibility complex (MHC) class II, it is unknown whether they present antigen directly to T cells *in vivo*. Through the combined efforts of ILC2 and DC, antigen-specific T helper type 2 (Th2) cells differentiate, and produce type 2 cytokines, further stimulating, recruiting and activating type 2 effector cells, as well as IL-2, activating and expanding ILC2 cells (blue arrows). Regulatory T cells (T_regs_) produce immunosuppressive cytokines such as IL-10 and transforming growth factor (TGF)-β, suppressing T cells and DC responses (blue solid line), and may also suppress ILC2 responses (blue dashed line). Helminths and their products are known to suppress through at least five key mechanisms (indicated in numbered red circles): (1) secretion of apyrases, enzymes which can degrade the inflammatory DAMP ATP to non-inflammatory adenosine monophosphate (AMP) 99,100; (2) secreted products which inhibit the release of IL-33 96; (3) a range of products suppress DC maturation to Toll-like receptor (TLR) signals 86–89; (4) secretions induce T_regs_ through the transforming growth factor (TGF)-β pathway 107; and (5) secreted enzymes degrade eotaxin, a chemokine required for eosinophil attraction 103.

Much interest is therefore focused on the cell type(s) involved in kick-starting the inflammatory response, in particular the prototypical antigen-presenting cell, the dendritic cell (DC) which, following infection, both takes up pathogen products for presentation to T cells and up-regulates a range of stimulatory molecules to drive T cell activation (Fig. 1). However, helminths can interfere dramatically with this critical process, suppressing the maturation of DCs following Toll-like receptor (TLR) ligation, reducing their production of inflammatory cytokines and reducing T cell responsiveness 86–88. In the case of schistosome egg antigen, this effect is due to omega-1, a glycoprotein which degrades intracellular host RNA, resulting in defective up-regulation of T cell-activating inflammatory cytokines such as IL-12 89. Some helminths may divert DC function entirely, as in the case of *H. polygyrus* infection, which expands a CD11c^lo^CD103^−^ dendritic cell phenotype which induces T_regs_ through retinoic acid release 90. Helminth inhibition of DCs is thus a potent method to block both initiation and recall of immune responses by naive or memory T cells.

As well as DCs, the recent recognition of a new category of immunocyte, the innate lymphoid cell (ILC), has expanded our knowledge of immune response initiators. While present in only small numbers, ILCs are capable of initiating type 2 allergy (in the case of the ILC2) 91 or type 1 colitis (in the case of ILC1/3 92). ILCs are activated by early innate cytokines such as IL-12 and IL-18 (ILC1); IL-25, IL-33 and TSLP (ILC2); and IL-1β and IL-23 (ILC3) 93. Their contribution forms part of an integrated sensory system reliant on epithelial and stromal cell signalling, which acts to raise the first alarm when the body is invaded by pathogens or subject to other traumas. Recent data show further that they can be integral to the initiation of T cell responses through provision of IL-13 and DC recruitment 91, as well as the amplification of T cell responses, as ILCs are activated through T cell provision of IL-2 94. Furthermore, several authors have established that ILCs can express class II major histocompatibility complex (MHC) and may thereby be able to present antigen to T cells 94,95. Thus, the ILC2 compartment presents an important target for helminth immunoregulation (Fig. 1).

A link between the ILC2 network and helminth immunomodulation has emerged very recently through the blockage of IL-33 production by secreted products of *H. polygyrus* (HES; Fig. 1); as IL-33 is a key driver of ILC2 activation, its loss thereby ablates the immediately subsequent ILC response required for induction and amplification of allergic responses 96. Furthermore, both *H. polygyrus* infection and HES induce the release of IL-1β from macrophages, which counter-regulates IL-25 and IL-33, resulting in diminished type 2 responses and greater susceptibility to chronic helminth infection 97. IL-33 itself is induced by the damage-associated molecular pattern (DAMP) extracellular adenosine triphosphate (ATP), which is released from stressed and damaged cells 98. Many parasite secretions, including HES, contain multiple apyrase enzymes 99,100 [which degrade inflammatory ATP to non-inflammatory adenosine monophosphate (AMP)], illustrating the multiple mechanisms through which helminths may interfere with DAMP, alarmin and ILC responses.

However, many questions remain as to the role of ILCs in long-term allergic conditions (such as steroid-resistant asthma), whether the ILC set-point is formed early in life and whether there are regulatory ILCs as well as inducers. The interdependence of T cell and ILC responses during initiation and recall of the immune response also remain to be elucidated: the potential role of ILC antigen presentation and the potential role of T_reg_ suppression on ILCs are as yet unclear (Fig. 1), and may provide further insights into the mechanisms of immune suppression used by helminth parasites.

Downstream of the induction process, whether through DCs and/or ILCs, the adaptive immune system is activated and amplified, in turn stimulating key innate effector cell populations such as neutrophils, macrophages and granulocytes 53. It appears that helminths are no less able to also modulate these innate populations. For example, both the dog hookworm *Ancylostoma caninum* and the livestock parasite *Haemonchus contortus* export factors which inhibit neutrophil activation 101,102. Moreover, human hookworm products also cleave eotaxin, suppressing recruitment of eosinophils 103. Alternatively activated macrophages differentiate in many helminth infection settings, and are able to both directly suppress bystander T cell proliferation 63 and to induce further forkhead box protein 3 (FoxP3^+^) T_reg_ differentiation 104. These type 2 macrophages mediate wound-healing effects 105 and quell inflammation in inflammatory conditions such as colitis 106.

## Forward look

How do we now re-evaluate the hygiene hypothesis in the light of the updated immunological picture? A number of new conclusions can be drawn that will advance the discussion of this stimulating concept. First, there is increasing focus on the initial ‘spark’ that ignites the allergic and inflammatory pathway. It seems likely that many infectious organisms have evolved means to suppress early ‘alarm’ signals, such as IL-33, and so may minimize the likelihood of a proinflammatory response being mounted.

Secondly, although this spark initiates a preliminary round of cellular reactions from innate cells such as monocytes and granulocytes, the response cannot take hold without positive feedback and amplification through signals and cytokines from the CD4^+^ T cell compartment. In this sense, T cells remain in control of the outcome of all immune responses, and promotion of anti-inflammatory regulatory T cells is likely to remain a keystone of any hygiene hypothesis formulation.

Thirdly, although effector T cell involvement supports a more vigorous, extensive and long-lived reaction, this inflammatory response is composed largely of innate cell types ranging from eosinophils and other granulocytes through to cells of the monocyte lineage. In many epidemiological instances of infections modulating pathology, underlying T cell phenotypes are not greatly altered but there is a powerful dampening of innate effector populations that deserves further investigation.

Finally, if our understanding of the hygiene hypothesis is to be translated into future therapies, these will largely need to be applied to patients in whom inflammatory diseases have already taken hold. Hence, a key factor is to consider the sustenance and ongoing aggravation of inflammatory responses, and how infectious agents and their molecular products can block or even reverse these pathways. In this manner the hygiene hypothesis would be both validated and transformed into a therapeutically valuable concept for future medicine.
